# Clinical Outcome among Nasopharyngeal Cancer Patients in a Multi-Ethnic Society in Singapore

**DOI:** 10.1371/journal.pone.0126108

**Published:** 2015-05-12

**Authors:** Han Wen Mak, Shan Hui Lee, Jeremy Chee, Ivan Tham, Boon Cher Goh, Siew Shuen Chao, Yew Kwang Ong, Kwok Seng Loh, Chwee Ming Lim

**Affiliations:** 1 Department of Otolaryngology—Head and Neck Surgery, National University Health System, Singapore, Singapore; 2 Department of Radiation Oncology, National University Health System, Singapore, Singapore; 3 Department of Hematology and Oncology, National University Health System, Singapore, Singapore; Central South University, CHINA

## Abstract

**Background:**

Nasopharyngeal cancer (NPC) is endemic among Chinese populations in Southeast Asia. However, the outcomes of non-Chinese NPC patients in Singapore are not well reported.

**Aim:**

To determine if non-Chinese NPC patients have a different prognosis and examine the clinical outcomes of NPC patients in a multi-ethnic society.

**Methods:**

Retrospective chart review of 558 NPC patients treated at a single academic tertiary hospital from 2002 to 2012. Survival and recurrence rates were analysed and predictive factors identified using the Kaplan-Meier method and Cox regression model.

**Results:**

Our cohort comprised 409 males (73.3%) and 149 females (26.7%) with a median age of 52 years. There were 476 Chinese (85.3%), 57 Malays (10.2%), and 25 of other ethnic groups (4.5%). Non-Chinese patients were more likely to be associated with advanced nodal disease at initial presentation (p = 0.049), compared with the Chinese. However, there were no statistical differences in their overall survival (OS) or disease specific survival (DSS) (p = 0.934 and p = 0.857 respectively). The 3-year and 5-year cohort OS and DSS rates were 79.3%, 70.7%, and 83.2%, 77.4% respectively. Advanced age (p<0.001), N2 disease (p = 0.036), N3 disease (p<0.001), and metastatic disease (p<0.001) at presentation were independently associated with poor overall survival. N2 disease (p = 0.032), N3 disease (p<0.001) and metastatic disease (p<0.001) were also independently associated with poor DSS. No predictive factors were associated with loco-regional recurrence after definitive treatment. Advanced age (p = 0.044), N2 disease (p = 0.033) and N3 disease (p<0.001) were independently associated with distant relapse.

**Conclusion:**

In a multi-ethnic society in Singapore, non-Chinese are more likely to present with advanced nodal disease. This however did not translate into poorer survival outcomes. Older patients with N2 or N3 disease are associated with a higher risk of distant relapse and poor overall survival.

## Introduction

Cancer of the nasopharynx is endemic in East Asia and South-East Asia [1, 2]. In Singapore, it is the 8^th^ most common cancer in males, with an age-standardized incidence of 9.5 per 100,000 per year [3]. This is in contrast to the United States and the rest of the world, with less than 1 case per 100,000 per year [4]. Ethnic differences in its etiology and presentation have been described. The keratinizing squamous cell carcinoma (WHO type 1) is more common among Caucasians in Western populations, while the EBV-associated undifferentiated variant (WHO type 2b) is seen predominantly among Chinese living in Southern China, Hong Kong, Taiwan, and Singapore [4–6]. Given its radiosensitive nature, chemo-radiation is the mainstay of treatment [7–10] with favorable overall 5-year survival rates of 75% to 83% [11, 12]. However, recurrent disease following definitive chemo-radiation occurs in approximately 10–15% of patients with distant metastases contributing to a poor outcome in these patients [13, 14].

The epidemiology and survival outcomes of nasopharyngeal carcinoma (NPC) are well described in Chinese populations. However, in the multi-ethnic society of Singapore, the epidemiology and the risk factors for loco-regional recurrence and survival of non-Chinese NPC patients are not well described. Specifically, whether non-Chinese patients constitute a distinct population with a different clinical outcome is unknown. Therefore, the aim of this study is to determine if non-Chinese NPC patients have a different clinical outcome. Our secondary aim is to identify predictive factors for survival and recurrence of NPC patients in a multi-ethnic patient population in Singapore.

## Materials and Methods

A retrospective chart review of clinical records was conducted and 586 NPC patients diagnosed at the National University Hospital, Singapore between January 2002 and October 2012 were identified. All patients who were treated and with follow-up data were included. Twenty-eight (4.8%) patients were excluded as they did not initiate or complete treatment at our center, leaving 558 (95.2%) patients for analyses. Patients’ follow-up was assessed up to December 2013. All information on patient demographics was obtained through patient clinical records, with approval from our local institutional review board (National Healthcare Group Ethics Committee Singapore; approval code 00418-AMD0002). Patient’s records and relevant information were anonymized and de-classified prior to analysis.

### Disease Diagnosis, Staging and Treatment

All patients were initially evaluated with history and physical examination. The disease diagnosis was confirmed histologically from biopsy of either the nasopharynx or its sites of metastases. All patients were staged according to the American Joint Committee on Cancer (AJCC) 7^th^ edition criteria [15]. The extent of local disease was determined either by computed tomography (CT) or magnetic resonance imaging (MRI) of the post-nasal space and neck. Assessment of distant metastasis was performed using either a combination of CT-thorax and abdomen and bone scan, or with a single whole body positron emission tomography–computed tomography (PET-CT) scan.

At our institution, all newly diagnosed NPC patients were presented at the head and neck cancer multi-disciplinary board comprising otolaryngologists, radiation and medical oncologists, radiologists and pathologists. The standard treatment protocol at our institution during the study period was to administer radiotherapy alone for early stage (Stage I and II) disease, with the addition of concurrent chemotherapy for late stage (Stage III, IVA, and IVB) disease. 3-D conformational radiotherapy (3-D CRT) or intensity-modulated radiotherapy (IMRT) were the primary radiotherapy techniques administered with IMRT being the standard of radiotherapy treatment from 2006. Platinum-based chemotherapy along with 5-fluorouracil (5-FU) was the primary chemotherapy regimen with a small cohort of stage IV patients receiving neo-adjuvant chemotherapy as a trial protocol.

### Classification of Ethnicity

For this study, ethnicity or race was determined by patients’ national identity records and retrieved from hospital registration records. As the Chinese comprised the majority of ethnicities, non-Chinese patients were grouped collectively for the purpose of analyses.

### Statistical Methods

We defined the starting point of all events as the date of disease diagnosis, and the times to the following endpoints were determined in months: disease specific survival (DSS—death due to NPC or treatment complications), overall survival (OS—death from any cause), loco-regional recurrence (LRR—recurrent disease in the nasopharynx or neck), and distant failure (DF—metastatic recurrence). Deaths due to intercurrent diseases, coroners’ cases, or unknown causes were considered censorings for OS. For the determination of 3-year and 5-year OS and DSS, only patients with 3 years and 5 years follow-up were considered respectively. In the assessment of disease recurrence, only patients with non-metastatic disease who received definitive treatment with curative intent were considered for analysis.

Statistical tests were performed using IBM SPSS (IBM SPSS Statistics for Windows, Version 20.0. Armonk, NY: IBM Corp.). Univariate analyses were performed using chi-square tests; ordinal chi-square tests were used to assess linearity in ordinal variables. Survival times were calculated using the Kaplan-Meier method and the differences were compared using log-rank tests. Multivariate analyses were performed using the Cox hazard regression model. A p-value of less than 0.05 was used to indicate statistical significance for all tests.

## Results

### Patient and Disease Characteristics

The mean age at diagnosis was 51.8 (range 14 to 94). There were 409 (73.3%) males and 149 females (26.7%). The distribution of ethnicity was: 476 Chinese (85.3%), 57 Malays (10.2%), and 25 of other ethnic groups (4.5%). Patients of other ethnicities include Indian (6, 1.1%), Caucasian (2, 0.4%), Kenyan (1, 0.2%) and others of mixed ethnicities (16, 2.9%). A majority of these cancers were the non-keratinizing undifferentiated WHO type 2b variant (95.5%) while the WHO type 2a variant (keratinizing undifferentiated) and WHO type 1 variant (squamous cell carcinoma) comprised the remaining 2.5% and 1.3% of our patient cohort respectively. We were unable to retrieve the histological reports of 4 (0.7%) patients, although the clinical records of diagnosis and treatment of NPC were evident in these patients. The median duration of follow-up was 41 months.

Our patient demographics and AJCC staging distribution are summarized in Table 1. In patients with M1 disease at diagnosis, the most common site of metastases was bone (34/53, 64.2%), followed by lung (20/53, 37.7%) and liver (15/53, 28.3%). Other sites accounted for 13.2% (7/53). These include eye (1), gastric/celiac lymph nodes (1), bone marrow (1), mediastinal/peritoneal lymph nodes (1), aortopulmonary lymph nodes (1), spleen (1) and adrenals (1).

**Table 1:** Summary of Patient and Disease Characteristics of Cohort.

Characteristic		n (total = 558)	%
**Age (median = 52)**	<20	3	0.5
	20–39	70	12.5
	40–59	359	64.3
	60–79	121	21.7
	≥80	5	0.9
**Gender**	Male	409	73.3
	Female	149	26.7
**Ethnicity**	Chinese	476	85.3
	Malay	57	10.2
	Others	25	4.5
**Histology**	*WHO 1	7	1.3
	WHO 2a	14	2.5
	WHO 2b	533	95.5
^**†**^ **AJCC Stage**	I	52	9.3
	II	137	24.6
	III	171	30.6
	IVA	90	16.1
	IVB	55	9.9
	IVC	53	9.5
**T classification**	T1	212	38.0
	T2	132	23.7
	T3	92	16.5
	T4	122	21.9
**N classification**	N0	121	21.7
	N1	173	31.0
	N2	189	33.9
	N3	75	13.4
**M classification**	M0	505	90.5
	M1	53	9.5

*World Health Organization

† American Joint Committee on Cancer

### Ethnic Differences

On univariate analysis, patients of non-Chinese ethnicity were more likely to be associated with higher nodal disease (p = 0.049), compared with those of Chinese ethnicity. In terms of age, gender, histology, AJCC stage and T classification, there was no significant difference between Chinese and non-Chinese. Given that Malays were the predominant non-Chinese cohort (57/82, 69.5%), a sub-analysis of comparison was performed. Similarly, Malays were more likely to present with a higher AJCC stage at diagnosis (p = 0.015), higher T and N disease (p = 0.033 and p = 0.016 respectively). Table 2 summarizes these characteristics.

**Table 2:** Comparison of Patient and Tumor Characteristics between Chinese versus non-Chinese in this NPC cohort.

Characteristic		Comparison between Chinese and non-Chinese			Comparison between Chinese and Malays	
		Chinese (n, %)	Non-Chinese (n, %)	*p-value*	Malays (n, %)	*p-value*
**Age**	<20	2 (0.4)	1 (1.2)	.*690*	1 (1.8)	.*862*
	20–39	63 (13.2)	7 (8.5)		3 (5.3)	
	40–59	300 (63.0)	59 (72.0)		43 (75.4)	
	60–79	106 (22.3)	15 (18.3)		10 (17.5)	
	≥80	5 (1.1)	0 (0.0)		0 (0.0)	
**Gender**	Male	348 (73.1)	61 (74.4)	.*809*	45 (78.9)	.*344*
	Female	128 (26.9)	21 (25.6)		12 (21.1)	
**Histology**	*WHO 1	5 (1.1)	2 (2.4)	.*591*	0 (0)	.*682*
	WHO 2a	13 (2.7)	1 (1.2)		1 (1.8)	
	WHO 2b	454 (95.4)	79 (96.3)		56 (98.2)	
^**†**^ **AJCC Stage**	I	46 (9.7)	6 (7.3)	.*419*	2 (3.5)	**.*015***
	II	121 (25.4)	16 (19.5)		9 (15.8)	
	III	146 (30.7)	25 (30.5)		18 (31.6)	
	IVA	76 (16.0)	14 (17.0)		12 (21.1)	
	IVB	42 (8.8)	13 (15.9)		10 (17.5)	
	IVC	45 (9.5)	8 (9.8)		6 (10.5)	
**AJCC Stage**	Early (I, II)	167 (35.1)	22 (26.8)	.*145*	11 (19.3)	**.*017***
	Late (III, IV)	309 (64.9)	60 (73.2)		46 (80.7)	
**T classification**	T1	182 (38.2)	30 (36.6)	.*237*	18 (31.6)	**.*033***
	T2	118 (24.8)	14 (17.1)		10 (17.5)	
	T3	78 (16.4)	14 (17.1)		9 (15.8)	
	T4	98 (20.6)	24 (29.3)		20 (35.1)	
**T stage**	Early (T1, T2)	300 (63.0)	44 (53.7)	.*107*	28 (49.1)	**.*041***
	Late (T3, T4)	176 (37.0)	38 (46.3)		29 (50.9)	
**N classification**	N0	108 (22.7)	13 (15.9)	**.*049***	7 (12.3)	**.*016***
	N1	149 (31.3)	24 (29.3)		15 (26.3)	
	N2	160 (33.6)	29 (35.4)		24 (42.1)	
	N3	59 (12.4)	16 (19.5)		11 (19.3)	
**N Involvement**	No (N0)	108 (22.7)	13 (15.9)	.*165*	7 (12.3)	.*071*
	Yes (Any N)	368 (77.3)	69 (84.1)		50 (87.7)	
**M classification**	M0	431 (90.5)	74 (90.2)	.*931*	51 (89.5)	.*795*
	M1	45 (9.5)	8 (9.8)		6 (10.5)	
**Total**		476	82		57	

*World Health Organization

† American Joint Committee on Cancer

### Overall and Disease Specific Survival

**Fig 1:**
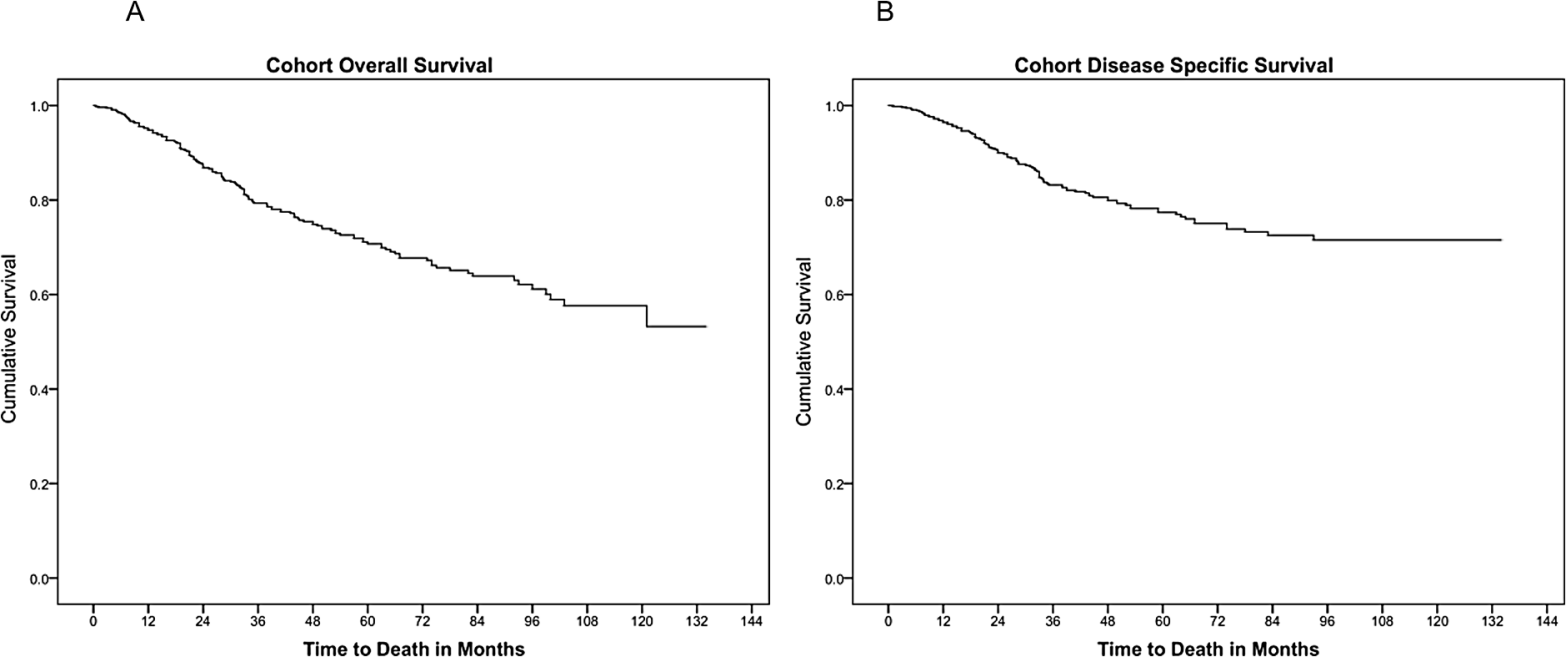
Cohort Survival Curves. (A) Overall survival. (B) Disease-specific survival. The five-year OS and DSS for Chinese and non-Chinese patients were 69.5% and 76.6%, 72.9% and 77.1% respectively, with no significant differences between the two groups (p = 0.934 and p = 0.857 respectively). Similarly, there was no difference in OS or DSS between the Chinese and Malays. (p = 0.640 and p = 0.898 respectively). These results are summarized in Table 3, and are represented by survival curves in Fig 2A and 2B.

The 3-year and 5-year overall survival (OS) rates of our cohort were 79.3% and 69.9%, while disease-specific survival (DSS) rates were 82.8% and 70.3% respectively. The mean OS was 96.1 months (95% CI 91.1–101.1) while the mean DSS was 106.6 months (95% CI 102.0–111.3). The survival curves for OS and DSS are shown in Fig 1A and 1B. The 3-year and 5-year OS for non-metastatic patients who received treatment with curative intent was 84.6% and 74.7% respectively, while 3-year and 5-year DSS was 88.3% and 81.5% respectively.

**Fig 2:**
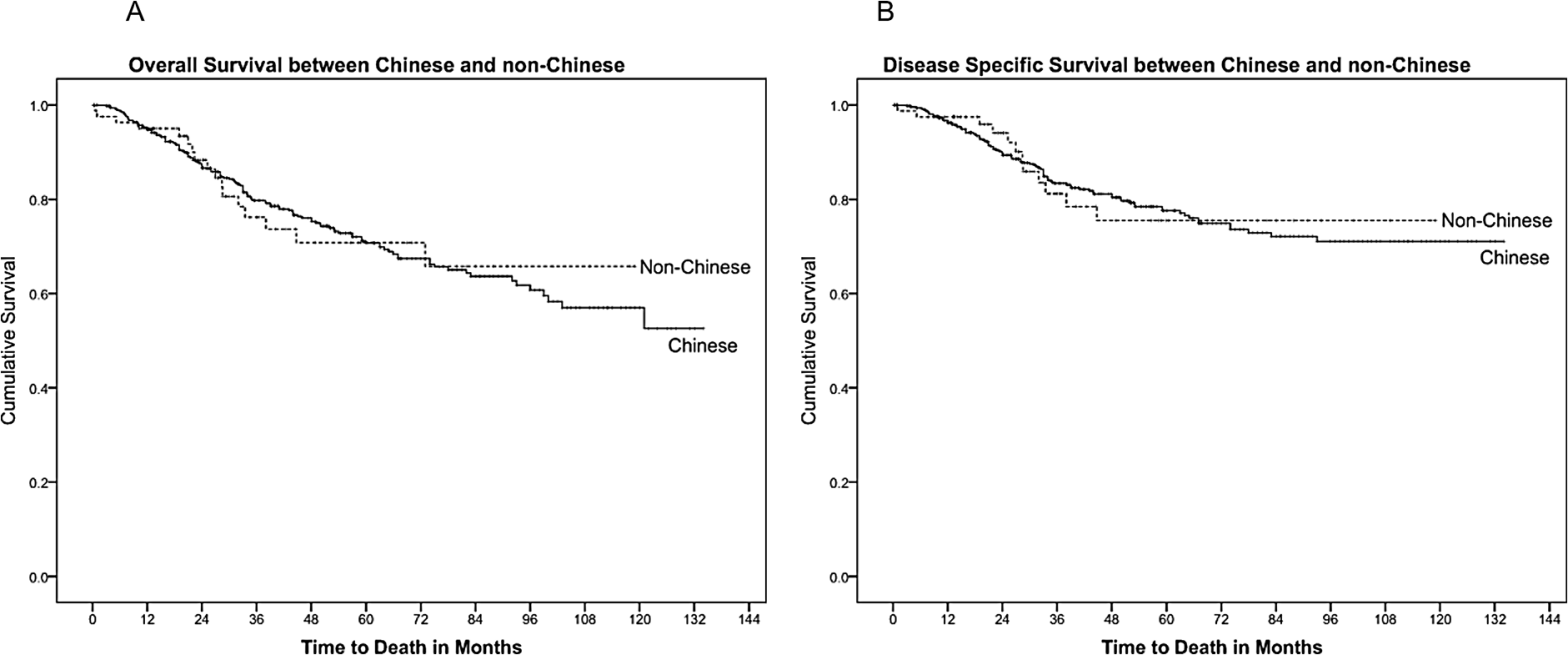
Survival Comparison between the Chinese and non-Chinese. (A) Overall survival. (B) Disease-specific survival.

**Table 3:** Cohort Overall Survival and Disease Specific Survival.

	Overall Survival, %				Disease Specific Survival, %			
	Cohort	Chinese	Non-Chinese	Malays	Cohort	Chinese	Non-Chinese	Malays
**3-year**	79.3	79.7	76.2	73.6	83.2	83.4	81.2	80.4
**5-year**	70.7	70.7	70.8	67.2	77.4	77.6	75.5	73.4

### Predictive Factors for Overall Survival and Disease Specific Survival

On univariate analysis, advanced age (p<0.001), higher T classification (p<0.001), higher N classification (p<0.001), and metastatic disease at presentation (p<0.001) were found to be significantly associated with poor OS, while patients with advanced age (p = 0.009), metastatic disease at presentation (p<0.001) and a higher T and N disease (p = 0.015 and p<0.001 respectively) were associated with poor DSS. These results are summarized in Table 4. Among non-Chinese patients, the presence of distant metastasis was the only factor associated with a poorer overall survival and disease specific survival.

**Table 4:** Univariate Analyses of Factors Associated with Overall Survival, Disease-Specific Survival, Loco-regional Recurrence and Distant Failure.

Characteristic		Overall Survival, p-value	Disease—Specific Survival, p-value	Loco-regional recurrence, p-value	Distant Failure, p-value
**Age**	<20	***<0*.*001***	***0*.*009***	*0*.*487*	*0*.*087*
	20–39				
	40–59				
	60–79				
	≥80				
**Gender**	Male	*0*.*123*	*0*.*332*	*0*.*914*	*0*.*615*
	Female				
**Race**	Chinese	*0*.*934*	*0*.*857*	*0*.*481*	*0*.*180*
	Non-Chinese				
	Chinese	*0*.*640*	*0*.*898*	*0*.*245*	*0*.*237*
	Malays				
**Histology**	*WHO 1	*0*.*169*	*0*.*470*	*0*.*158*	*0*.*404*
	WHO 2a				
	WHO 2b				
^**†**^ **AJCC Stage**	I	***<0*.*001***	***<0*.*001***	*0*.*254*	***<0*.*001***
	II				
	III				
	IVA				
	IVB				
	IVC				
**T classification**	T1	***<0*.*001***	***0*.*015***	*0*.*231*	*0*.*212*
	T2				
	T3				
	T4				
**N classification**	N0	***<0*.*001***	***<0*.*001***	*0*.*329*	***<0*.*001***
	N1				
	N2				
	N3				
**M classification**	M0	***<0*.*001***	***<0*.*001***		
	M1				

*World Health Organization

† American Joint Committee on Cancer

On multivariate analysis, age was significantly associated with worse OS (Hazard Ratio = 1.028 for each additional year, p<0.001, 95% CI 1.013–1.044). Among the disease factors, N2 disease (H.R. 1.75, p = 0.036, 95% CI 1.04–2.96), N3 disease (H.R. 2.99, p<0.001, 95% CI 1.69–5.27) and metastatic disease at presentation (H.R. 5.54, p<0.001, 95% CI 3.59–8.57) were independently associated with poor OS. Independent factors associated with poorer DSS were N2 disease (H.R. 2.13, p = 0.032, 95% CI 1.07–4.24), N3 disease (H.R. 3.75, p<0.001, 95% CI 1.79–7.88) and metastatic disease (H.R. 8.70, p<0.001, 95% CI 5.30–14.27) at presentation.

The mean OS and DSS for patients with metastatic disease was 28.2 months (95% CI 19.3–37.1), and 31.2 months (95% CI 20.9–41.6) respectively, while the mean OS and DSS for patients with non-metastatic N3 disease was 74.2 months (95% CI 59.2–89.2) and 85.6 (95% CI 71.1–100.8) months respectively. Table 5 summarizes the results of multi-variate analysis of factors associated with poorer OS and DSS.

**Table 5:** Multi-variate Analysis of Factors Associated with a Poorer Overall Survival and Disease-Specific Survival.

		Overall Survival				Disease-Specific Survival			
Covariate		*p-value*	Hazard Ratio	95% Confidence Interval		*p-value*	Hazard Ratio	95% Confidence Interval	
				Lower	Upper			Lower	Upper
**Age**	Each additional year	***<0*.*001***	1.028	1.013	1.044	*0*.*157*	1.013	0.995	1.031
**Gender**	Male								
	Female	*0*.*747*	0.937	0.630	1.393	*0*.*620*	1.124	0.707	1.788
**Race**	Chinese								
	Non-Chinese	*0*.*647*	0.886	0.529	1.486	*0*.*781*	0.916	0.495	1.696
**T-classification**	T1	*0*.*354*				*0*.*725*			
	T2	*0*.*537*	0.858	0.527	1.396	*0*.*470*	1.225	0.707	2.123
	T3	*0*.*438*	1.210	0.747	1.962	*0*.*272*	1.388	0.773	2.490
	T4	*0*.*223*	1.314	0.847	2.039	*0*.*422*	1.253	0.723	2.170
**N-classification**	N0	*<0*.*001*				*0*.*004*			
	N1	*0*.*598*	1.157	0.672	1.992	*0*.*110*	1.771	0.879	3.568
	N2	***0*.*036***	1.752	1.038	2.957	***0*.*032***	2.125	1.066	4.239
	N3	***<0*.*001***	2.986	1.692	5.269	***<0*.*001***	3.751	1.786	7.879
**M-classification**	M0								
	M1	***<0*.*001***	5.542	3.585	8.566	***<0*.*001***	8.698	5.302	14.270

### Loco-Regional Recurrence and Distant Failure

Five hundred and five (505) patients underwent definitive therapy and were considered for analysis of disease recurrence. With a median follow-up duration of 46 months, 93 patients (18.4%) experienced loco-regional recurrence (LRR) and 84 (16.6%) distant failure (DF). The median time to LRR or DF was 18.0 (95% CI 14.782–21.218) and 14.6 (95% CI 10.831–18.436) months respectively. Patients who developed loco-regional recurrence underwent surgical salvage (nasopharyngectomy or neck dissection) when appropriate. Patients with LRR had a median survival of 83 months (95% CI 63.498–102.502) compared to 34.7 months in patients with DF (95% CI 27.413–41.987, p<0.001).

### Predictive Factors for Loco-Regional Recurrence and Distant Failure

On both univariate and multi-variate analysis, there were no predictive factors associated with LRR. Ethnicity was not a risk factor for developing LRR. There was no difference in the LRR rate between the Chinese and non-Chinese (p = 0.481).

On univariate analysis, a higher N disease (p<0.001) was associated with an increased risk of distant failure after primary treatment. On multivariate analysis, age (H.R. 1.02 for each additional year, p = 0.044, 95% CI 1.001–1.04), N2 disease (H.R. 2.31, p = 0.017, 95% CI 1.16–4.60) and N3 disease (H.R. 5.41, p < 0.001, 95% CI 2.59–11.33) were independently associated with distant failure.

Subgroup analysis within the N3 group of patients revealed a higher risk of distant failure in the N3b group compared with the N3a group (H.R. 2.790, 95% CI 1.097–7.092, p = 0.031). The median time to distant failure for N3b and N3b patients was 16.0 months and 19.0 months respectively (p = 0.399). Table 6 summarizes the factors associated with LRR and distant failure.

**Table 6:** Multi-variate Analysis of Factors Associated with a Higher Risk of Loco-regional Recurrence and Distant Failure.

		Loco-Regional Recurrence				Distant Failure			
Covariate		*p-value*	Hazard Ratio	95% Confidence Interval		*p-value*	Hazard Ratio	95% Confidence Interval	
				Lower	Upper			Lower	Upper
**Age**	Each additional year	*0*.*656*	1.004	0.986	1.023	***0*.*044***	1.021	1.001	1.043
**Gender**	Male								
	Female	*0*.*839*	1.048	0.667	1.646	*0*.*703*	1.102	0.670	1.810
**Race**	Chinese								
	Non-Chinese	*0*.*627*	1.153	0.649	2.050	*0*.*564*	1.182	0.670	2.087
**T-classification**	T1	*0*.*436*				*0*.*682*			
	T2	*0*.*974*	1.009	0.580	1.756	*0*.*395*	1.286	0.721	2.295
	T3	*0*.*552*	1.202	0.655	2.208	*0*.*473*	1.268	0.663	2.426
	T4	*0*.*130*	1.523	0.883	2.626	*0*.*243*	1.418	0.789	2.547
**N-classification**	N0	*0*.*520*				*<0*.*001*			
	N1	*0*.*861*	0.950	0.535	1.686	*0*.*571*	1.243	0.586	2.635
	N2	*0*.*292*	1.340	0.778	2.308	***0*.*014***	2.389	1.197	4.770
	N3	*0*.*891*	0.944	0.415	2.148	***<0*.*001***	5.451	2.602	11.418

## Discussion

Nasopharyngeal carcinoma is an endemic cancer among Chinese in Southern China and Southeast Asia. Unsurprisingly, our study showed a predominance of Chinese in our NPC cohort (85.3%). Genetic and environmental factors have been implicated in the pathogenesis of NPC in Chinese [16, 17]. This has been illustrated in epidemiological studies where Chinese from Southern China and Hong Kong who migrated to the United States of America were found to have a similarly high incidence of NPC compared to their indigenous population, but with decreasing risks with each successive generation born in the United States [18]. Genome-wide association studies have also supported a genetic basis with the HLA region being identified as an at-risk locus among Chinese populations [19].

In the multi-ethnic society of Singapore, the epidemiology of NPC among non-Chinese is not well defined. Our study demonstrated that the non-Chinese comprised 14.7% of our cohort of NPC patients, with the Malays being the predominant ethnic group (69.5%) developing this disease in our non-Chinese cohort. Interestingly, the endemic non-keratinizing undifferentiated WHO type 2b variant (EBV-associated) was predominant in all ethnic groups suggesting that environmental factors may account for this observation [20]. Another explanation could be attributed to genetic cofactors such as a history of intermarriage between the Malay indigenous population and Chinese ancestors in Southeast Asia [17]. However, this hypothesis could not be confirmed given the retrospective nature of this study. Certainly, this genetic basis may occur since specific HLA haplotypes have been identified among nasopharyngeal carcinoma patients [21]. Despite being a low-risk ethnic group for cancer in Singapore, the increasing incidence of cancer including nasopharyngeal carcinoma among the Malay population is a cause of concern necessitating further research into this phenomenon [22].

A more concerning finding was that Malay patients presented with a more advanced primary and nodal disease at presentation than the Chinese. This observation may be attributed to a lower index of suspicion of NPC among referring primary care practitioners since NPC is traditionally thought of as a disease afflicting mainly the Chinese. Additionally, differences in cultural attitudes and disease awareness among ethnicities may also contribute to a delayed presentation. This cultural bias of poorer awareness was demonstrated among non-Chinese patients who presented with a more advanced breast cancer in Singapore [23, 24]. This study serves to highlight the need for increased awareness of NPC in non-Chinese patients among general practitioners in our population.

Despite Malays presenting with a more advanced disease, their survival and disease control rates were not significantly different when compared to the Chinese following definitive therapy. Given that the EBV-associated WHO type 2b variant is ubiquitously found in both Chinese and non-Chinese, it is likely that the radiosensitive nature of this variant portends a favorable prognosis even when detected at a more advanced stage. Certainly, a type II error may also account for this observation due to the small sample size of the non-Chinese population (57/558, 10.2%). Larger scale multi-center studies may be necessary to overcome this limitation.

Previous studies have reported that age, male gender, and advanced T and N disease were poor prognostic factors for NPC [14, 25–29]. In our series, age, advanced N disease and metastatic disease at presentation were associated with a poorer outcome. Similar observations have also been described by Lu *et al*. where an advanced N disease was the predominant predictive factor of poor outcome [29]. It is possible that with advances in radiotherapy techniques and experience, a high loco-regional control rate is achievable and hence, a more advanced T disease may no longer be as important a prognostic factor as it used to be. However, a temporal comparison of outcomes between patient cohorts will be required to validate this hypothesis.

Similarly, we did not find any predictors of loco-regional recurrence (LRR) in our cohort compared to previous studies in which age and late T disease were significant predictors [25, 27, 30, 31]. As these studies were conducted in the late 1990s and early 2000s, advances in radiotherapy techniques may have accounted for the improved loco-regional control rate seen in our study.

Lastly, a more advanced nodal status (N2 and N3 disease) was associated with an increased risk of distant failure. This finding was consistent with previous studies [14, 28, 29, 31]. Specifically, patients with N3 nodal disease had the highest risk of distant failure after primary chemo-radiation. Additionally, patients with N3b disease are at an even higher risk of distant failure compared to those with N3a disease. Whatever the mechanisms of distant failure, the outcome of this group of patients remains poor. These results reflect how distant failure still remains the primary cause of treatment failure and mortality, even while local disease control has improved with advances in radiotherapy techniques and successful surgical salvage [32]. Based on this observation, neoadjuvant chemotherapy and other novel strategies should be investigated in patients with N3 disease in order to minimize the risk of distant relapse.

## Conclusion

In a multi-ethnic society in Singapore, non-Chinese are more likely to present with higher nodal disease than the Chinese. This however does not translate into poorer survival outcomes. N2 or N3 disease is associated with a higher risk of distant relapse and poor overall survival. This group of patients may benefit from systemic or targeted therapy which needs to be addressed in prospective clinical studies.
